# Nicotinic Acetylcholine Receptor Alpha6 Contributes to Antiviral Immunity via IMD Pathway in *Drosophila melanogaster*

**DOI:** 10.3390/v16040562

**Published:** 2024-04-03

**Authors:** Zhiying Wang, Xiaoju Lin, Wangpeng Shi, Chuan Cao

**Affiliations:** Department of Entomology, China Agricultural University, Beijing 100193, China; zhiyingwang@cau.edu.cn (Z.W.); xiaojulin@yeah.net (X.L.)

**Keywords:** nicotinic acetylcholine receptors, antiviral immunity, insecticide, IMD pathway

## Abstract

Currently, insecticides that target nicotinic acetylcholine receptors (nAChR) are widely used. Studies on the sublethal effects of insecticides have found that they can affect the amount of virus in insects. The mechanism by which insecticides affect insect virus load remain unclear. Here, we show that nAChR targeting insecticide can affect viral replication through the immune deficiency (IMD) pathway. We demonstrate that a low dose of spinosad (6.8 ng/mL), acting as an antagonist to *Drosophila melanogaster* nicotinic acetylcholine receptor α6 (Dα6), significantly elevates Drosophila melanogaster sigmavirus (DMelSV) virus titers in adults of *Drosophila melanogaster*. Conversely, a high dose of spinosad (50 ng/mL), acting as an agonist to Dα6, substantially decreases viral load. This bidirectional regulation of virus levels is absent in *Dα6*-knockout flies, signifying the specificity of spinosad’s action through *Dα6*. Furthermore, the knockdown of *Dα6* results in decreased expression of genes in the IMD pathway, including *dredd*, *imd*, *relish*, and downstream antimicrobial peptide genes *AttA* and *AttB*, indicating a reduced innate immune response. Subsequent investigations reveal no significant difference in viral titers between *relish* mutant flies and *Dα6*-*relish* double mutants, suggesting that the IMD pathway’s role in antiviral defense is dependent on *Dα6*. Collectively, our findings shed light on the intricate interplay between nAChR signaling and the IMD pathway in mediating antiviral immunity, highlighting the potential for nAChR-targeting compounds to inadvertently influence viral dynamics in insect hosts. This knowledge may inform the development of integrated pest management strategies that consider the broader ecological impact of insecticide use.

## 1. Introduction

The insect nicotinic acetylcholine receptor, a pentamer consisting of five identical or different subunits, plays a pivotal role in mediating the transmission of acetylcholine between synapses and is a vital target for insecticides [1]. In 2018, approximately 29% of the insecticide market was dedicated to products targeting nicotinic acetylcholine receptors, with neonicotinoid-based insecticides accounting for 24% of this category, making them one of the most widely used insecticides at present [2].

Recent studies have found that neonicotinoid insecticides influence the viral load in insect hosts. Italian bees infected with chronic bee paralysis virus (CBPV) were found to have significantly higher load when exposed to high concentrations of imidacloprid compared to a control group [3]. Similarly, bees exposed to sublethal doses of thiamethoxam and imidacloprid for 30 days displayed a significant increase in deformed wing virus (DWV) levels [4]. However, the precise mechanism by which neonicotinoid insecticides impact virus replication is not yet understood.

There is evidence suggesting that nicotinic acetylcholine receptors (nAChRs) may interact with insect immune pathways, thereby influencing the host’s defense against pathogens. Exposure of *Drosophila melanogaster* to thiamethoxam resulted in the up-regulation of leucine-rich repeat (LRR), a negative regulator of NF-κB signaling, which in turn suppressed the NF-κB signaling pathway [4]. Additionally, a study has shown that the expression of antimicrobial peptide gene *drosomycin* in *D. melanogaster nicotinic acetylcholine receptor α7* (*Dα7*) mutant line was significantly reduced compared to the control line following infection with *Pseudomonas lutea* [5]. Expression of antimicrobial peptide genes is regulated by the Toll pathway and the immune deficiency (IMD) pathway, which are part of inducible humoral immunity signaling pathways. Previous research has shown that the IMD pathway is involved in antiviral immunity. For instance, *PGRP-LC* mutant strains exhibited significantly higher viral load of Drosophila C virus (DCV) compared to wild-type flies post infection [6]. Moreover, viral titers of both DCV and Solenopsis invicta virus (SINV) were substantially increased in the gut of *relish* mutant strains [6]. Some antimicrobial peptides can act as antiviral effectors, reducing the expression of the *Drosophila* antimicrobial peptide genes *DptB* and *AttC*, both leading to a significant increase in SINV viral load in *D. melanogaster* [7]. Drosophila melanogaster sigmavirus (DMelSV) belongs to the family *Rhabdoviridae*, which includes viruses that infect the vertebrate central nervous system such as Rabies virus [8]. DMelSV also infects insect nervous systems and sometimes causes paralysis upon exposure to CO_2_ [9]. In the present study, we found that an insecticide that targets nAChR, spinosad, significantly alters the replication of a nervous system infecting virus, DMelSV (*Rhabdoviridae*), in *D. melanogaster*, indicating the interaction between insect nervous and immune systems. Further experiments showed that the DMelSV virus load in the host was influenced by *D. melanogaster nicotinic acetylcholine receptor α6* (*Dα6*) through the modulation of antimicrobial peptides AttA and AttB via the IMD pathway. We also demonstrated that this *Dα6*-regulated immunity can apply broadly to a variety of RNA viruses. In summary, our study revealed a new mechanism by which an acetylcholine receptor can regulate host humoral immunity and engage in the dynamics of host–virus interactions.

## 2. Materials and Methods

### 2.1. Flystocks

Fly stocks were maintained at 25 °C, 12 h light/12 h dark and 65% humidity. The standard medium consisted of maize flour, sugar, agar powder, yeast, butyl paraben, and water.

Fly stocks used are listed in Table 1.

BL67048 was crossed with BL80510 to obtain the *Dα6-*overexpression strain *P* {*TOE.GS01969*} *attP40*/ *P*{*UAS*-*3xFLAG*.*dCas9*.*VPR*} *attP40*; *P*{*tubP*-*GAL4*} *LL7*. The control line *P*{*GS00089*} *attP40*/ *P*{*UAS*-*3xFLAG*.*dCas9*.*VPR*} *attP40*; *P*{*tubP*-*GAL4*} *LL7* was generated by crossing BL67048 and BL67539. *Daughterless*-Gal4 was crossed with V52342 to obtain knock-down line *daughterless*-Gal4; *AttB*-RNAi, and the progeny *daughterless*-Gal4; a cross between *daughterless*-Gal4 and V60000 was used as the control.

The *Dα6*-KO strain and *w^1118^*; *Rel^E20^e^s^* were crossed with *if*/*cyo*; *MKRS*/*TM6B, Tb*, respectively, to obtain the strains *Dα6^KO^*/*cyo*; +/*TM6B*, *Tb* and +/*cyo*; *Rel^E20^e^s^*/*TM6B, Tb*. Then *Dα6^KO^*/*cyo*; +/*TM6B, Tb* and +/*cyo*; *Rel^E20^e^s^*/*TM6B, Tb* were crossed to obtain *Dα6^KO^*/*cyo*; *Rel^E20^e^s^*/*TM6B, Tb*. Eventually, a pure double mutant line *Dα6^KO^*; *Rel^E20^e^s^* was obtained by self-crossing this line. PCR was carried out to verify the presence or absence of *relish* and *nAChRα6*. DNA was extracted by Chelex following the manufacturer’s instructions. The PCR reaction consisted of incubation at 98 °C (5 min), followed by 30 cycles of 98 °C (10 s), 55 °C (30 s), and 72 °C (1 min), then 72 °C (5 min). And PCR products were run on 1.5% agarose gels.

Primers used for PCR are listed in Table 2.

### 2.2. Virus Isolates

DCV, hap23 strain of DMelSV (hap23sv) were kindly provided by Professor Francis Jiggins’s lab. To ensure the crude extract of virus obtained after grinding was mainly DMelSV, eggs of a susceptible strain, 22a, were immersed in 70% ethanol for 2 min, 10% sodium hypochlorite solution for 10 min, and immersed in distilled water 3 times, each time for 2 min, to remove other viruses attached to the surface of the fly eggs. Then hap23sv was injected into hatched 22a females. After 20 days, the CO_2_ sensitivity test was performed, and about 1000 adult flies carrying the virus were selected to extract DMelSV using the protocol described in Magwire et al., 2011 [11].

Reovirus, galbut virus, and La Jolla virus were extracted from wild flies collected in Shanghai, Penglai, Qinghai, and the West Campus of China Agricultural University. After grinding wild flies to obtain the crude virus extract [11], the RNA was extracted for qPCR tests for different drosophila viruses using primers published in Webster C. L’s paper [12]. It was found that the crude virus extract contained reovirus, galbut virus, La Jolla virus, and other viruses. Then, the crude virus extract was diluted to 10^7^, 10^8^, 10^9^, and 10^10^ times, and used to inoculate S2 cells. RNA was extracted from different inoculations for qPCR inspection to detect wells that only contain single virus species. S2 cells with a single virus were enriched for extraction of purified virus isolates. 

### 2.3. Immune Challenge of Adult Flies

Adult females 2–6 days after eclosion were injected with 69 nl of the virus suspension using NanojectⅢ (Drummond, 3-000-207, Broomall, PA, USA) intra-abdominally, as described in detail in references [13].

The gram-positive *Lactococcus lactis* (Testobio, TS414132, Zhejiang, China) was precultured in GM17 medium at 30 °C with 200 rpm shaking. The log phase of bacterial culture was spun down and the resulting pellet resuspended in GM17 to achieve OD_600_ = 1 (Thermofisher, Beijing, China), while the gram-negative *Serratia marcescens* (Testobio, TS343044, Zhejiang, China) was precultured in LB medium at 26 °C with 120 rpm shaking. The log phase of bacterial culture was spun down and the resulting pellet resuspended in LB to achieve OD_600_ = 0.1. Stainless steel needles (FST, 26002-15, 0.15 mm, Guangdong, China) dipped into the suspension were used to pierce the lateral side of the thorax of a pre-anaesthetized adult female. Dry spore powder of *Beauveia bassiana* was bought from Green Hengfeng (Guangxi, China). Adult females anesthetized under hypothermia at 4 °C were shaken for 1 min in a petri dish containing *Beauveria*. After being well-covered with spores, flies were transferred back to their vials. In these experiments, butyl p-hydroxybenzoate was not added to the corn flour medium.

Infected adult females were incubated at 25 °C, while the over-expression strains were maintained at 27 °C and the RNAi strains were maintained at 29 °C to enhance Gal4 expression. Flies were sampled for quantitative rt-PCR at 2, 3, 6, 12 days after inoculation. To assay for susceptibility to bacteria and fungi, we recorded survival every 24 h until all the flies had died.

### 2.4. Quantitative RT-PCR

Total RNA was extracted from 10–20 individuals from each line using TRIzol (ambion, Beijing, China) following the manufacturer’s instructions. RNA was reverse transcribed into cDNA using PrimeScript™ RT reagent Kit (Takara, Beijing, China). Gene expression analysis was performed by qRT-PCR using TB Green^®^ Premix Ex Taq™ II FAST qPCR (Takara, Beijing, China), according to the manufacturer’s protocol in the CFX Connect Real-Time PCR Detection System (Bio-Rad, Beijing, China) through the Bio-Rad Manager ™ Software Version 3.1. Relative mRNA expression levels were normalized to that of Rpl32. 

Primers used for qPCR are listed in Table 3. The primers for galbut virus and reovirus were designed by this study. The primers for galbut virus and La Jolla virus were from Webster C.L [12], and the primers for DCV were from Chuan Cao [14]. The primers for hap23sv were obtained from Chi-Wei Tsai [15].

### 2.5. Quantify the Bacterial Load

After 24 h, infected flies were anesthetized and collected in 1.5 mL microcentrifuge tubes and store tubes on ice. Each tube had 10 flies. To determine the bacterial load post infection, we used the protocol described in Kuo et al. 2012 [16]. Firstly, 400 μL of LB or GM17 liquid medium was added to each tube and ground for 30 s at 60 HZ using a fully automatic sample rapid grinding instrument (Shanghai Jingxin Industrial Development, JXFSTPRP-24L, Shanghai, China). The ground homogenate was diluted at 1:1000, and 100 μL of diluent were applied to each LB or GM17 solid medium. After placing the medium in an incubator at 37 °C for 12 h, photographs of colonies were taken and colony counts were performed using ImageJ.

### 2.6. RNA-seq Analysis

Transcriptomic profiling was performed using three independent biological replicates per feeding. Female W^1118^(control) and nAChR α6-attP (KO line) were injected with hap23sv. After 6 days, flies were collected for RNA extraction using TRIzol (Ambion, Beijing, China). The concentration and quality of the extracted total RNA were estimated using Nanodrop (IMPLEN, Westlake Village, CA, USA) and Agilent 2100 Bioanalyzer, respectively. For cDNA library preparation, the library concentration was analyzed by Agilent 2100 Bioanalyzer (Agilent Technologies, Santa Clara, CA, USA). Sequencing was performed using Illumina HiSeq sequencer (NEB, Ipswich, MA, USA). Hisat v2.0 (Dallas, TX, USA) was used to map clean reads to the reference *D. melanogaster* genome. Htseq-count, with gene location information from Mapped Reads, was used to quantify transcript expression levels. DESeq2 was used for differential expression analysis between sample groups to identify the set of differentially expressed genes between the two biological conditions. Fold change represents the expression ratio between the two sample groups. The false discovery rate (FDR) was obtained by correcting for the difference in significance *p*-value. The selection criteria for detecting differentially expressed genes were a fold change of 2 or greater and an FDR of less than 0.05. Gene Ontology Resource was used for functional classification. TPM (Transcripts Per Million) was an index of transcript expression level. 

### 2.7. Insecticide Dilution and Exposure

Spinosad (MedChemExpress, Shanghai, China) was diluted by methanol to create 6.8 ng/mL and 50 ng/mL solution. To investigate whether spinosad targeting *Dα6* affects the quantity of DMelSV, wild-type flies infected with DMelSV were placed in a media tube supplemented with methanol, 6.8 ng/mL, and 50 ng/mL spinosad, respectively. The feed tubes containing spinosad were changed every other day. After 6 days, the flies were tested for viral load. 

### 2.8. Effect of Antimicrobial Peptide AttA Provisioning on Viral Load

To evaluate the effect of AttA on the viral load of DMelSV, two groups of one-week old adult females were set up. After 3 days of infection, 5 μM antimicrobial peptide AttA (AlpaLifeBio, Shenzhen, China) dissolved in Tris buffer (20 mM Tris, 150 mM NaCl, pH = 8.0) was added to the surface of fly feed in the experimental group. To ensure uniform coverage of AttA on the surface, 35 μL of AttA suspension were dropped-added to each tube. Each experimental group consisted of four replicates, each containing 15 flies. The control groups received the fly feed supplemented with only Tris buffer. After 6 days, the amount of virus in flies were detected by qPCR.

### 2.9. Statistical Analyses

All results are expressed as the mean ± standard deviation (SE). The Shapiro–Wilk test was used for checking normality of data and homoscedasticity was checked by using the F test. Statistical analyses of results between two treats at the same time point were carried out using an independent-Sample *t*-test, where multiple treats were compared by a one-way ANOVA.

To examine the survival of adult flies infected by bacteria and fungi, the Kaplan–Meier method was used for statistical analysis, while data were compared by the log-rank Mantel–Cox test.

## 3. Results

### 3.1. Spinosad and Its Target Dα6 Can Affect DMelSV Replication

**Figure 1:**
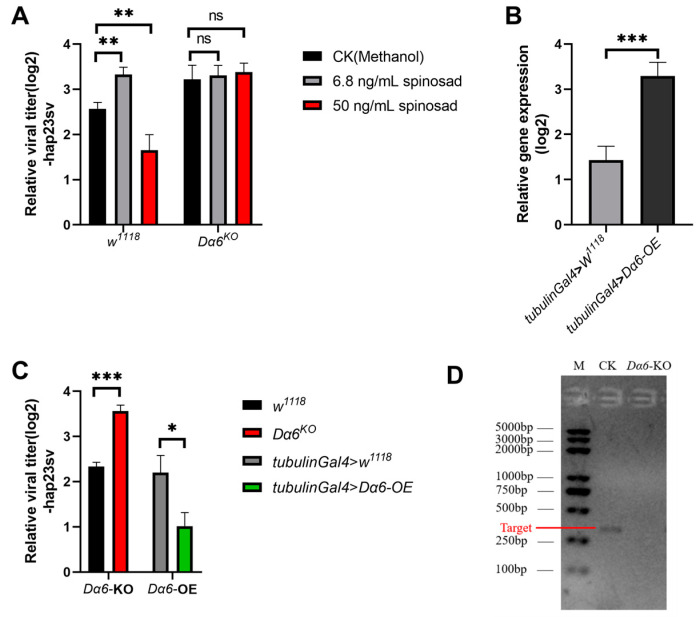
Effect of spinosad and *Drosophila melanogaster nicotinic acetylcholine receptor α6* (*Dα6*) on the amount of Drosophila melanogaster sigmavirus (DMelSV) in the host. (**A**) The amounts of virus in vivo in wild-type flies (*w^1118^*) and the *Dα6* knockout strain infected with DMelSV after 6 days of feeding methanol (control), 6.8 ng/mL, and 50 ng/mL spinosad. (**B**) In vivo, *Dα6* was significantly increased in flies by the tub-gal4-driven *Dα6* overexpression line. (**C**) The difference in the amount of DMelSV in the *Dα6* knockout strain compared with wild-type flies (*w^1118^*) and the difference in the amount of DMelSV in the *Dα6* overexpressed strain compared with control flies. (**D**) Agarose gel electrophoresis confirmed the absence of *Dα6* expression in *Dα6* knockout line. More than five replicates were performed for each experiment. Statistical significance was determined via the *t*-test (ns, non-significant, * *p* < 0.05, ** *p* < 0.01, and *** *p* < 0.001). Error bars represent the standard errors.

Spinosad is known to bind to the *Drosophila* acetylcholine receptor Dα6, acting as an agonist to activate the cation permeation channel of the receptor. Recent studies have shown that at low doses, spinosad can antagonize Dα6 [17]. Our findings showed that flies fed with low concentrations of spinosad exhibited a significantly higher DMelSV virus load, while flies fed with high concentrations of spinosad showed a significantly lower virus load compared to the control group (Figure 1A). These observations suggest that different concentrations of spinosad have opposite impacts on DMelSV virus load in *D. melanogaster*, potentially due to its dual role as an agonist and antagonist of Dα6. 

To further investigate the role of *Dα6* in regulating DMelSV virus titer in the host, we infected a *Dα6* knockout strain and a strain with tub-gal4 driven *Dα6* whole-body overexpression with DMelSV, to examine whether *Dα6* can affect viral replication. Results showed a significant increase in the viral load in *Dα6* knockout flies compared to the wild type (Figure 1C). In contrast, the amount of DMelSV in the *Dα6* overexpression strain was significantly lower than that in the control (Figure 1C). We then fed DMelSV-infected *Dα6* knockout flies with 6.8 ng/mL or 50 ng/mL of spinosad, respectively. Six days after treatment, the viral load in flies fed different concentrations of spinosad did not significantly differ from those fed with methanol as controls (Figure 1A). These results suggest that *Dα6* negatively regulates the load of DMelSV and that spinosad’s effect on viral replication is dependent on the presence of *Dα6*.

### 3.2. Knockdown of Dα6 Negatively Regulates the Expression of IMD Pathway Genes

To explore the mechanism by which *Dα6* affects DMelSV virus load in the host, we performed RNA-seq analysis on wild-type (*w^1118^*) and *Dα6* knockout flies after they were infected with DMelSV for 6 days. 

**Figure 2:**
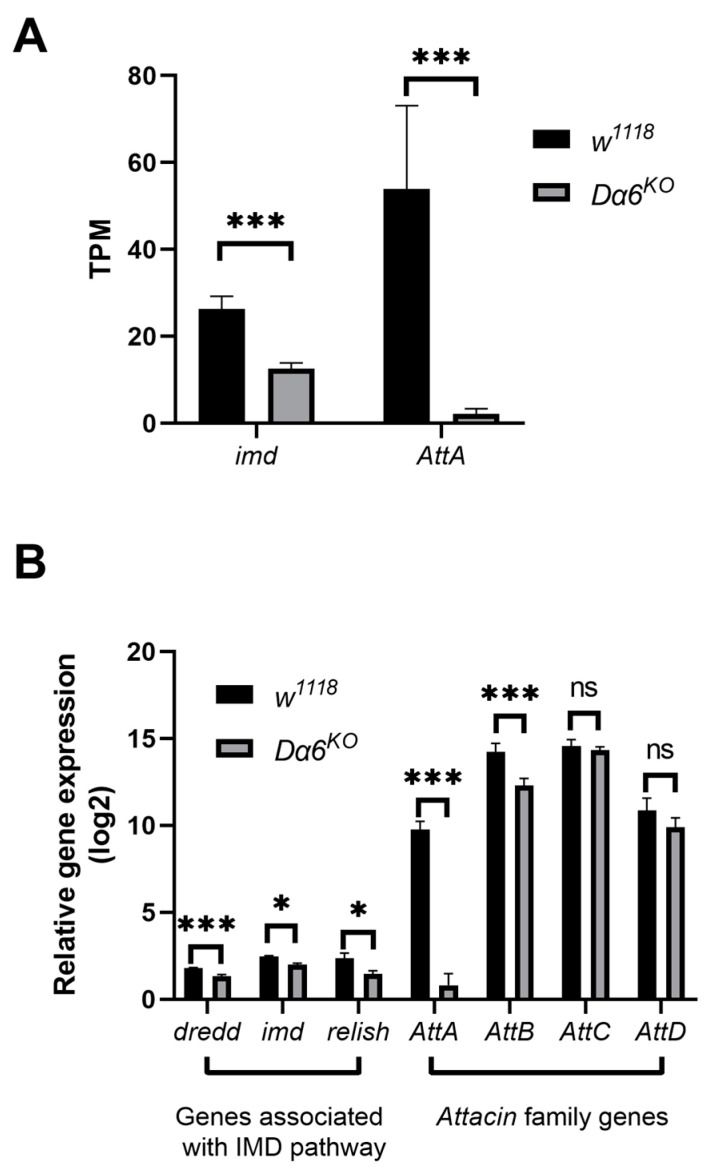
Knockout of *Dα6* significantly reduced the expression of genes involved in the immune deficiency (IMD) pathway. (**A**) TPM of genes involved in the IMD pathway in wild-type flies (*w^1118^*) and *Dα6*-knockout flies at 6 days after infection with DMelSV. (*n* = 3) (**B**) Differences in the expression of *imd*, *Dredd*, and *Attacin* family genes in the gene Dα6 knockout strain flies compared with wild-type flies (*w^1118^*) at 6 days after infection with DMelSV and difference in *relish* expression in gene *Dα6*-knockout flies compared with wild-type flies (*w^1118^*) at 12 days after infection with DMelSV. More than five replicates were performed for each experiment. Statistical significance was determined via the *t*-test (ns, non-significant, * *p* < 0.05, and *** *p* < 0.001). Error bars represent the standard errors.

After knocking-out *Dα6*, the differential genes were mainly involved in iron ion binding, membrane formation, and metabolic processes (Figure S1), which was consistent with the major functions of *Dα6*, cation transport, synaptic transmission, and muscle homeostasis. Interestingly, the knockout of *Dα6* resulted in significant changes in the expression of immune genes against gram-negative bacteria, such as *imd*, *AttA*, and *TotA*. As a result, we screened for differentially expressed genes that were mainly involved in the immunity of gram-negative bacteria and we found that genes associated with IMD pathway, including *imd*, and downstream antimicrobial peptide genes *AttA*, exhibited significant down-regulation in *Dα6* knockout flies compared to wild-type flies (Figure 2A). RT-qPCR were performed on a separate set of experimental samples confirmed the RNAseq findings (Figure 2B). Furthermore, expression of *relish*, a key transcription factor in the IMD pathway, was found to be significantly reduced in *Dα6* knockout flies (Figure 2B). These results indicate that *Dα6* significantly affects the expressions of genes in IMD pathway and its downstream amp genes in flies. 

### 3.3. Dα6 Regulation of Host Immunity against DMelSV via IMD Pathway

**Figure 3:**
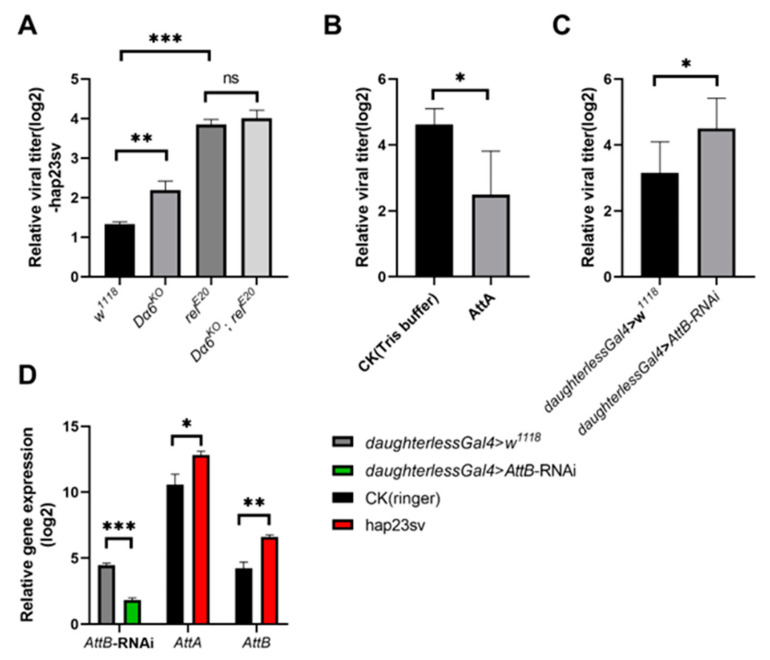
*Dα6* participated in the host immune process against DMelSV through the IMD pathway. (**A**) Differences in viral load in wild-type flies, *Dα6* and *relish* single mutant flies, and double mutant flies 6 days after DMelSV infection. (**B**) Differences in the amount of DMelSV in the flies fed AttA and Tirs buffer 6 days after infection with DMelSV. (**C**) Difference in the amount of DMelSV in *AttB*-RNAi strain compared with control flies. (**D**) Difference in the expression of *AttA* and *AttB* in wild-type flies 6 days after DMelSV injection versus ringer injection. In vivo, *AttB* was significantly decreased in flies by the *daughterless*-gal4-driven *AttB*-RNAi line. More than four replicates were performed for each experiment. Statistical significance was determined via the *t*-test and one-way ANOVA (ns, non-significant, * *p* < 0.05, ** *p* < 0.01, and *** *p* < 0.001). Error bars represent the standard errors.

To investigate if the *Dα6*-mediated immunity is achieved through the regulation of the IMD pathway, we constructed a double mutant strain of *Dα6* and *relish*. Single mutant of *Dα6* and *relish*, double mutant flies, and wild-type controls were injected with DMelSV and the viral load was measured after 6 days. We observed that knocking out *Dα6* or *relish* gene knockout both led to a significant increase in the titers of DMelSV compared to the wild-type fly (*w^1118^*) (Figure 3A). Interestingly, viral load in in the *Dα6* and *relish* double mutant flies was not significantly different from that in the *relish* single knockout strain (Figure 3A). These results suggest that the *Dα6*-mediated antiviral effect is dependent on *relish* gene.

We then directly investigated the role of the IMD pathway and its key genes in the immunity against DMelSV. Since the expression of antimicrobial peptide gene AttA and AttB were significantly decreased when knocking out *Dα6*, we first investigate the antiviral role of these two genes. We synthesized AttA and fed it to DMelSV-infected flies and a significantly decreased viral load was observed compared to the control group (Figure 3B). Because antimicrobial peptide AttB was not successfully synthesized, we instead knocked down *AttB* gene via RNAi. Knocking down *AttB* significantly increased viral load in flies (Figure 3C). In addition, the expression of the antimicrobial peptide genes AttA and AttB, downstream of the IMD pathway, was significantly induced by DMelSV infection (Figure 3D). These results suggest that antimicrobial peptides AttA and AttB of the IMD pathway play a significant role in *D. melanogaster*’s immunity against DMelSV. 

### 3.4. Dα6 Plays a Role in a Broad Antiviral Immunity against Variety of RNA Viruses

**Figure 4:**
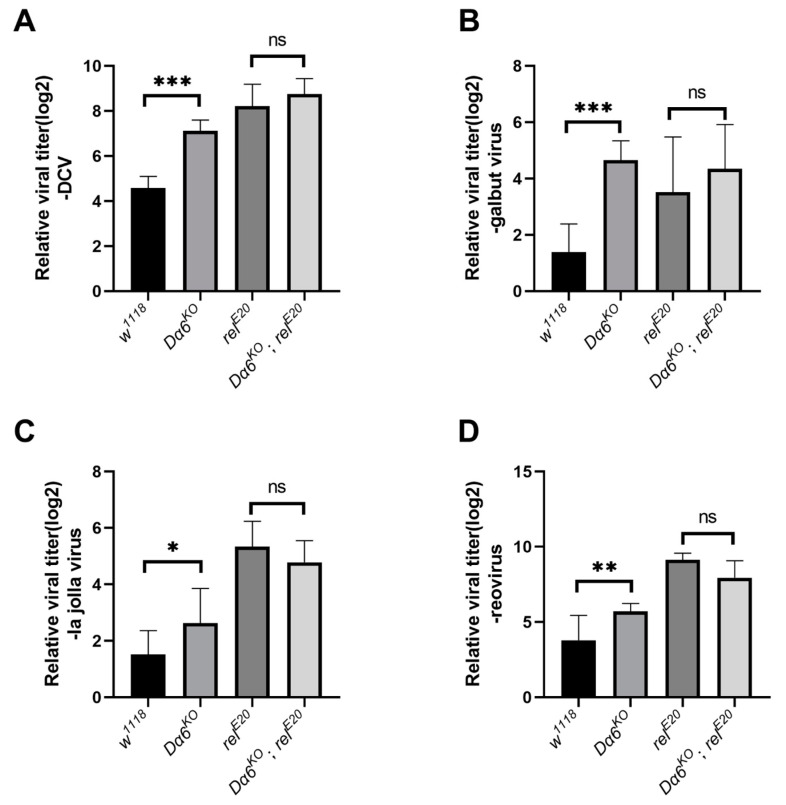
*Dα6* participated in the host immune process against a variety of RNA viruses through *relish*. (**A**) Differences in viral load in wild-type flies, *Dα6* and *relish* single mutant flies, and double mutant flies 2 days after DCV infection. (**B**) Differences in viral load in wild-type flies, *Dα6* and *relish* single mutant flies and double mutant flies 12 days after galbut virus infection. (**C**) Differences in viral load in wild-type flies, *Dα6* and *relish* single mutant flies and double mutant flies 3 days after La Jolla virus infection. (**D**) Differences in viral load in wild-type flies, *Dα6* and *relish* single mutant flies, and double mutant flies 6 days after reovirus infection. More than five replicates were performed for each experiment. Statistical significance was determined via the one-way ANOVA (ns, non-significant, * *p* < 0.05, ** *p* < 0.01, and *** *p* < 0.001). Error bars represent the standard errors.

To determine if the *Dα6*-mediated antiviral mechanism extends to other viruses, we tested four RNA viruses from different families: DCV (*Dicistroviridae*), galbut virus (*Partitiviridae*), La Jolla virus (LJV, *Iflaviridae*), and Reovirus (*Reoviridae*). We measured the viral load in wild-type, single mutants of *Dα6* and *relish,* and double mutant flies at specific time points post-infection with each virus: 2 days for DCV, 12 days for galbut virus, 3 days for La Jolla virus, and 3 days for reovirus. The results demonstrated that the viral loads for all tested viruses were significantly higher in *Dα6* knockout flies than in the controls. Meanwhile, no significant differences in the viral load were observed between the *Dα6* and *relish* double mutant strain and the *relish* single knockout strain, consistently across all tested RNA viruses (Figure 4A–D). This indicates that the Dα6-mediated viral immunity via relish is a broad-spectrum mechanism applicable to various RNA viruses.

### 3.5. Dα6 Does Not Contribute to Immunity to Bacteria or Fungi

**Figure 5:**
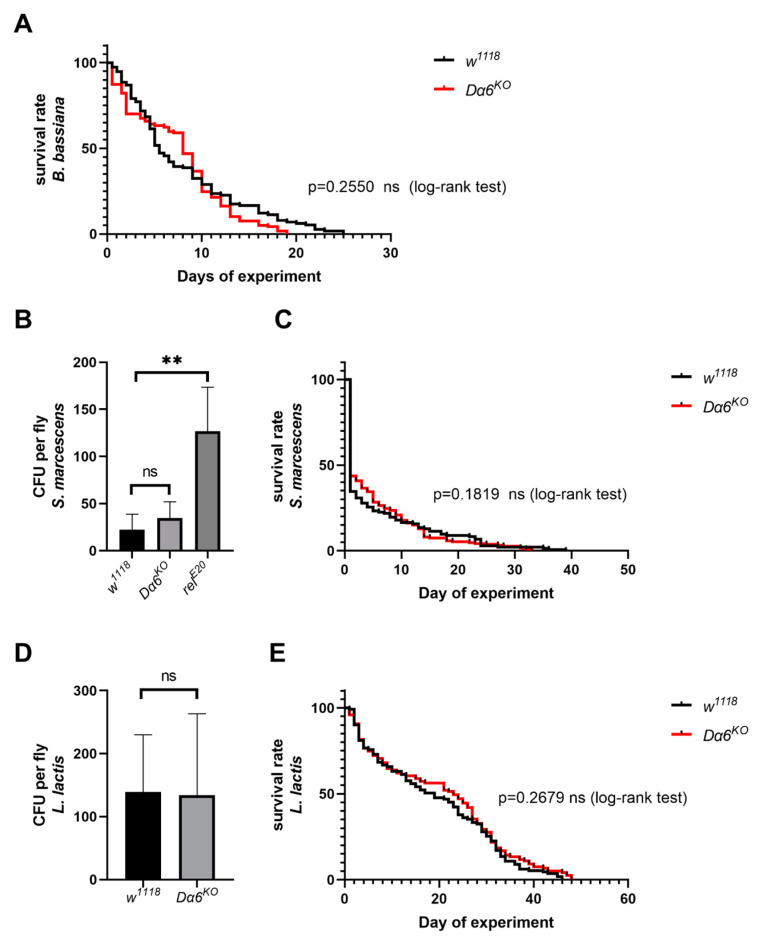
*Dα6* had no immune effect on some other pathogens. (**A**) The survival rate of wild-type flies and *Dα6* knockout flies after infection with *B. bassiana*. (log-rank test). (**B**) Differences in the bacterial load in wild-type flies, *Dα6* and *relish* single mutant flies 1 day after infection with *S. marcescens*. (**C**) Survival rate of wild-type flies and *Dα6* knockout flies 1 day after infection with *S. marcescens* (log-rank test). (**D**) Differences in the bacterial load in wild-type flies, *Dα6* and *relish* single mutant flies 1 day after infection with *L. lactis*. (**E**) Survival rate of wild-type and *Dα6* knockout flies 1 day after infection with *L. lactis* (log-rank test). More than four replicates were performed for each experiment. Statistical significance was determined via the *t*-test (ns, non-significant, ** *p* < 0.01). Error bars represent the standard errors.

To verify whether *Dα6* conferred immunity is general to IMD pathway regulated pathogens, the *Dα6* mutant strains were tested with virous fungus and gram-negative bacteria: *B. bassiana*, *S. marcescens*, and *L. lactis*, respectively. We counted the daily death rate of *Dα6* knockout and wild-type flies after exposure to *B. bassiana* and plotted the survival function curve. We found no significant difference in the survival ability of *Dα6* knockout flies infected with *B. bassiana* compared to the control group (Figure 5A). Additionally, one day after inoculation with *S. marcescens*, there was no significant difference in the amount of bacteria found in flies with the *Dα6* knockout line compared to the control group (Figure 5B). The daily mortality rate of *Dα6* knockout and wild-type flies infected with *S. marcescens* were not significant different. (Figure 5C). Similar results were also observed for gram-positive bacteria *L. lactis* (Figure 5D,E). This indicates that the knockout of *Dα6* did not significantly affect the amount of bacteria in flies or its ability to survive compared to the control group. In conclusion, the data suggest that *Dα6* may not exert a broad immune effect against other IMD-regulated bacterial or fungal pathogens.

## 4. Discussion

The involvement of nAChRs in host–virus interactions in insects has been relatively unexplored. Our study shed light on the role of Dα6 in the antiviral immunity *D. melanogaster* against a variety of RNA viruses. A series of experiments showed that this Dα6-mediated immunity is effective via modulating the key genes in IMD pathway. These results not only expand our understanding of Dα6’s role within insect immunity but also provide insights into the broader implications of nAChR-mediated pathways in host–pathogen dynamics. 

The nAChR in *Drosophila melanogaster* contains seven alpha subunits (α1, α2, α3, α4, α5, α6, α7) and three beta subunits (β1, β2, β3) [18]. Dα5, Dα6, and Dα7 were grouped into a single cluster with similar subunits found in anopheles, honeybee, bombyx, drosophila, and trig labra [19] and are all homologs to human α7 nicotinic acetylcholine receptor (hAChRα7). Previous studies have shown that *hAChR*α7 plays an essential role in response to virus infections [20]. Giordani et al. (2023) found that down-regulation of *Dα7* in flies infected with *Pseudomonas luteosus* led to a significant decrease in the expression of *drosomycin*, an antifungal gene mainly regulated by the Toll pathway [5]. These results suggest that Dα6 and Dα7, two subunits with high amino acid sequence similarity, may have an impact on the level of expression of AMPs. However, the mechanisms of how nAChRs modulate the expression of AMPs remain unclear. 

A previous study demonstrated that higher levels of reactive oxygen species (ROS) caused by spinosad are only observed in the presence of Dα6 [17], and in GO analysis, *Dα6* was involved in regulating the activities of many enzymes, especially oxidation-reduction process and oxidoreductase activity (Figure S1). Interestingly, it has been shown that increased ROS levels in flies leads to significant up-regulation of AMP *Drs* and *Dpt* expression levels in adipocytes, and *Relish* may act genetically as a master regulator of intestinal ROS-induced global AMP responses in adipocytes [21]. This implies that the immune process regulated by *Dα6* in the IMD pathway may be achieved by affecting oxidation–reduction process.

The RNAi pathway mediated by small interfering RNA (siRNA) is the most broad-spectrum antiviral mechanism in insects, and it is involved in the immunity of flies to DCV, SINV and other viruses [22,23]. However, we did not find any significant RNAi pathway-related gene changes caused by *Dα6* mutant in RNAseq. When we examined the expression of *Dcr2*, a key gene in the RNAi pathway by q-PCR, we found that there was no significant difference between the *Dα6*-KO line and the control (Figure S2). At present, some immune mechanisms such as Toll pathway, IMD pathway, JAK-STAT pathway, and a variety of cellular immunity such as cell phagocytosis and apoptosis have been proved to be involved in insect antiviral immunity [24]. AMPs have been identified as important effectors of antimicrobial and antifungal responses in the Toll and IMD pathways in flies [25]. At the same time, AMPs also could display potent antiviral activity [26]. AMPs such as melittin can inhibit cell-associated production of human immunodeficiency virus 1 (HIV-1) by suppressing HIV-1 gene expression [27]. Analysis of the effect of melittin on cell-associated virus production revealed lower levels of HIV-1 mRNAs [27]. Interestingly, treatment of C6/36 cells with different concentrations of Cecropin-D peptides limited infection with dengue virus [28]. It is evidenced that AMPs may exert a direct antiviral effect. However, the mechanisms by which these proteins interfere with the virus in *D. melanogaster* remain unclear. The broader implications of our findings also touch upon the impact of insecticides on insect pathogen susceptibility. There are observations that insects become more vulnerable to its natural pathogens following exposure to insecticides. *Bombyx mori* was more susceptible to *Enterobacter cloacae* sp. when exposed to sublethal doses of phoxim [29]. *Escherichia coli*-infected *Enallagma cyathigerum* larvae exposed to 0.5 μg/L chlorpyrifos had a tenfold higher bacterial load than unexposed larvae [30], while a study by Wang et al. (2023) found that *Aedes aegypti* treated with 10 ng/mL spinetoram during the larval stage had significantly higher amounts of dengue virus after becoming adults than the control [31]. Our study contributes to this topic by demonstrating that the insecticide spinosad-targeting Dα6 influences many RNA viruses load through interference with the IMD pathway. 

In light of these discoveries, future assessments of insecticide deployment should take into account the potential for these substances to inadvertently alter the pathogenic landscape within insect populations. Our research provides a stepping stone for further exploration into the intricate relationship between insecticide use and the immune competency of insects, thereby informing strategies for pest management that are both effective and ecologically considerate.

## Figures and Tables

**Table 1:** Fly stocks.

Gene Type	Stock ID	Source
w*; *P{UAS*-*3xFLAG.dCas9.VPR} attP40*; *P{tubP-GAL4} LL7*/*T(2;3)TSTL14*, *SM5*:*TM6B*, *Tb^1^*	BL67048	Bloomington Drosophila Stock Center, Bloomington, IN, USA
*if*/*cyo*; *daughterless*Gal4	TB00153	Tsinghua Stock Center, Beijing, China
*Dα6* ^KO^	DRZ17-CG4128-1	CCT related Drosophila line generated by Dr. Yi Rao’s Lab at Peking University, Beijing, China [10]
*w^1118^*	V60000	Vienna Drosophila Resource Center, Vienna, Austria
*AttB*-RNAi	V52342	Vienna Drosophila Resource Center, Vienna, Austria
y^1^sc^*^v^1^sev^21^; *P{TOE.GS01969}attP40*	BL80510	Bloomington Drosophila Stock Center, Bloomington, IN, USA
y^1^sc^*^v^1^sev^21^; *P{GS00089} attP40*	BL67539	Bloomington Drosophila Stock Center, Bloomington, IN, USA
*w^1118^*; *Rel^E20^e^s^*	BL9457	Bloomington Drosophila Stock Center, Bloomington, IN, USA
*if*/*cyo*; *MKRS*/*TM6B*, *Tb*	-	Obtained from Genetics Department, University of Cambridge, Cambridge, United Kingdom
-	22a (DMelSV susceptible strain)	Obtained from Genetics Department, University of Cambridge, Cambridge, United Kingdom

**Table 2:** Primers used for PCR.

Name	Sequence 5′→3′
KO-α6-F1	GCAATCGCATGAAGGAGCTG
KO-α6-R1	CTATCCACAACCATTGCCGC
KO-REL-F1	CCGAAAACCATGGAACTGCA
KO-REL-R1	TAGCAACGCCGAAACTAACG

**Table 3:** Primers used for qPCR.

Name	Sequence 5′→3′
RpL32 qRT-PCR F	TGCTAAGCTGTCGCACAAATGG
RpL32 qRT-PCR R	TGCGCTTGTTCGATCCG-TAAC
SIGMAV 1343F	ATGTAACTCGGGTGTGACAG
SIGMAV 1496R	CCTTCGTTCATCCTCCTGAG
Relish-qF	AACTAACTTGGACAGCCCACATTC
Relish-qR	TTTGCGTATGCCTCCCACACGGTTTC
Attacin A F	CAATGGCAGACAATCTGG
Attacin A R	ATTCCTGGGAAGTTGCTGTG
Attacin B F	ACAACAATGCTGGTCATGGTGCC
Attacin B R	ATGGGCCTCCTGCTGGAAGACA
Attacin C F	TTGGACAAGTGTTCGCAGCGGG
Attacin C R	TCCAGGCCGTGTCCATGATTGT
Attacin D F	GTCACTAGGGTTCCTCAG
Attacin D R	GCCGAAATCGGACTTG
imd-qF	TTCGGCTCCGTCTACAACTT
imd-qR	GTGATCGATTATGGCCTGGT
dredd-qF	ACATTGCCCTTCTCCACAGA
dredd-qR	CATGGCGATGCTGTTGGATG
DCV_qPCR_599_F	GACACTGCCTTTGATTAG
DCV_qPCR_733_R	CCCTCTGGGAACTAAATG
IflaB_qPCR_4F_928	GCTCTGATATCCCGGCC
IflaB_qPCR_4R_1064	GCAGCTTTTGAACCATATTGTG
Dmel-Reo 900F1	AAGGGAAAGAGGCGGCTCGTAT
Dmel-Reo 900R1	GTTGTTCTGGCGACGGCAGTT
pGal-but-qpcr-F1	CGACAAGGAATACAGCATACCA
pGal-but-qpcr-R1	CTTCATCAGCGGATTCAGCATA
nAChRα6qF	GTGGAACGACTACAATCTGCG
nAChRα6qR	AAGATACCAGGGGGCACGTA
Dcr-2-F	GCTTTTATGTGGGTGAACAGGG
Dcr-2-R	GGCTGTGCCAACAAGAACTT

## Data Availability

The data presented in this study are available upon request from the corresponding authors.

## Supplementary Materials

The following supporting information can be downloaded at: https://www.mdpi.com/1999-4915/16/4/562/s1, Figure S1: Functional classification of GO in RNA-seq, Figure S2: Difference in the expression of *Dcr2* in the gene Dα6 knockout strain flies compared with wild-type flies (*w^1118^*) at 6 days after infection with sigma virus.
