# Correction: Wang et al. Nicotinic Acetylcholine Receptor Alpha6 Contributes to Antiviral Immunity via IMD Pathway in *Drosophila melanogaster*. *Viruses* 2024, *16*, 562

**DOI:** 10.3390/v16050742

**Published:** 2024-05-08

**Authors:** Zhiying Wang, Xiaoju Lin, Wangpeng Shi, Chuan Cao

**Affiliations:** Department of Entomology, China Agricultural University, Beijing 100193, China; zhiyingwang@cau.edu.cn (Z.W.); xiaojulin@yeah.net (X.L.)

## Error in Table

In the original publication [1], there was a mistake in “Table 1. Fly stocks” as published. The fly stock BL67048 rather than TB00129 was used in our study. But it was written incorrectly in the writing process of this article. 

The corrected “Table 1. Fly stocks” appears below. 

**Table 1:** Fly stocks.

Gene Type	Stock ID	Source
w*; *P{UAS*-*3xFLAG.dCas9.VPR} attP40*; *P{tubP-GAL4} LL7*/*T(2;3)TSTL14*, *SM5*:*TM6B*, *Tb^1^*	BL67048	Bloomington Drosophila Stock Center, Bloomington, IN, USA
*if*/*cyo*; *daughterless*Gal4	TB00153	Tsinghua Stock Center, Beijing, China
*Dα6* ^KO^	DRZ17-CG4128-1	CCT related Drosophila line generated by Dr. Yi Rao’s Lab at Peking University, Beijing, China [10]
*w^1118^*	V60000	Vienna Drosophila Resource Center, Vienna, Austria
*AttB*-RNAi	V52342	Vienna Drosophila Resource Center, Vienna, Austria
y^1^sc^*^v^1^sev^21^; *P{TOE.GS01969}attP40*	BL80510	Bloomington Drosophila Stock Center, Bloomington, IN, USA
y^1^sc^*^v^1^sev^21^; *P{GS00089} attP40*	BL67539	Bloomington Drosophila Stock Center, Bloomington, IN, USA
*w^1118^*; *Rel^E20^e^s^*	BL9457	Bloomington Drosophila Stock Center, Bloomington, IN, USA
*if*/*cyo*; *MKRS*/*TM6B*, *Tb*	-	Obtained from Genetics Department, University of Cambridge, Cambridge, United Kingdom
-	22a (DMelSV susceptible strain)	Obtained from Genetics Department, University of Cambridge, Cambridge, United Kingdom

## Text Correction

There was an error in the original publication [1]. BL67048 and y^1^sc^*^v^1^sev^21^; *P* {*TOE*.*GS01969*} *attP40* can be crossed to obtain a *Dα6*-overexpression line, while TB00129 could not obtain it.

A correction has been made to “2. Materials and Methods “, “2.1. Flystocks”, “Paragraph 3”:

BL67048 was crossed with BL80510 to obtain the *Dα6-*overexpression strain *P* {*TOE.GS01969*} *attP40*/ *P*{*UAS*-*3xFLAG*.*dCas9*.*VPR*} *attP40*; *P*{*tubP*-*GAL4*} *LL7*. The control line *P*{*GS00089*} *attP40*/ *P*{*UAS*-*3xFLAG*.*dCas9*.*VPR*} *attP40*; *P*{*tubP*-*GAL4*} *LL7* was generated by crossing BL67048 and BL67539. *Daughterless*-Gal4 was crossed with V52342 to obtain knock-down line *daughterless*-Gal4; *AttB*-RNAi, and the progeny *daughterless*-Gal4; a cross between *daughterless*-Gal4 and V60000 was used as the control.

The authors state that the scientific conclusions are unaffected. This correction was approved by the Academic Editor. The original publication has also been updated.
