# Supplementary material for: Nicotinic Acetylcholine Receptor Alpha6 Contributes to Antiviral Immunity via IMD Pathway in Drosophila melanogaster
Source: Viruses. 2024 Apr 3;16(4):562. doi: 10.3390/v16040562 (PMC11054842; doi:10.3390/v16040562)
Supplement: Supplementary file 1 [file viruses-16-00562-s001.zip › viruses-2927960-supplementary.pdf]

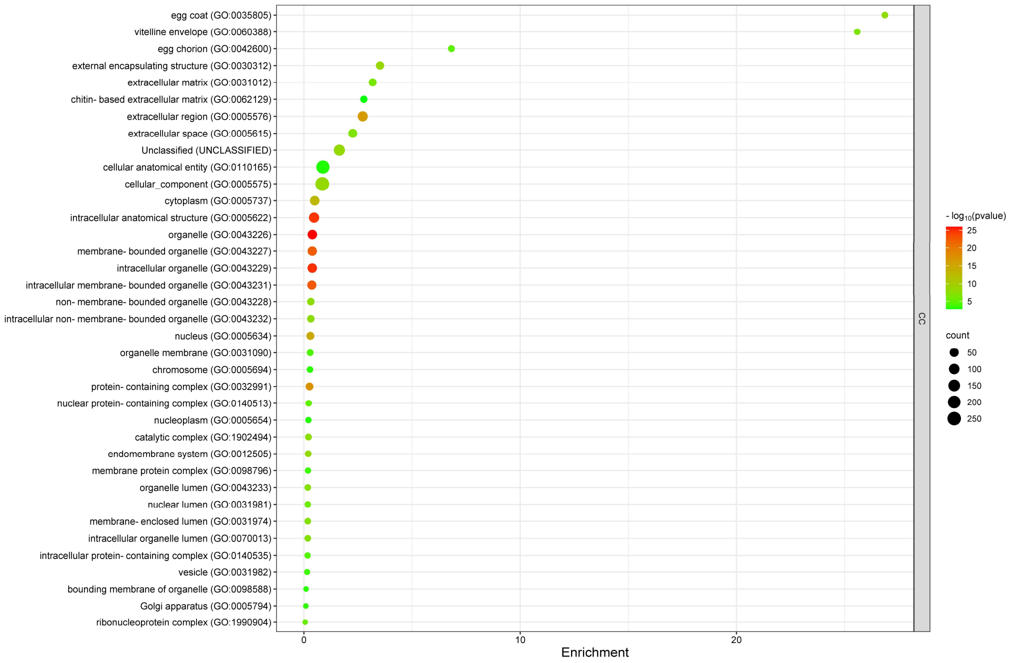
egg coat (GO:0035805)
vitelline envelope (GO:0060388)
egg chorion (GO:0042600)
external encapsulating structure (GO:0030312)
extracellular matrix (GO:0031012)
chitin- based extracellular matrix (GO:0062129)
extracellular region (GO:0005576)
extracellular space (GO:0005615)
Unclassified (UNCLASSIFIED)
cellular anatomical entity (GO:0110165)
cellular_component (GO:0005575)
cytoplasm (GO:0005737)
intracellular anatomical structure (GO:0005622)
organelle (GO:0043226)
membrane- bounded organelle (GO:0043227)
intracellular organelle (GO:0043229)
intracellular membrane- bounded organelle (GO:0043231)
non- membrane- bounded organelle (GO:0043228)
intracellular non- membrane- bounded organelle (GO:0043232)
nucleus (GO:0005634)
organelle membrane (GO:0031090)
chromosome (GO:0005694)
protein- containing complex (GO:0032991)
nuclear protein- containing complex (GO:0140513)
nucleoplasm (GO:0005654)
catalytic complex (GO:1902494)
endomembrane system (GO:0012505)
membrane protein complex (GO:0098796)
organelle lumen (GO:0043233)
nuclear lumen (GO:0031981)
membrane- enclosed lumen (GO:0031974)
intracellular organelle lumen (GO:0070013)
intracellular protein- containing complex (GO:0140535)
vesicle (GO:0031982)
bounding membrane of organelle (GO:0098588)
Golgi apparatus (GO:0005794)
ribonucleoprotein complex (GO:1990904)
0
10
20
Enrichment
CC
- log10(pvalue)
25
20
15
10
5
count
50
100
150
200
250

(A)

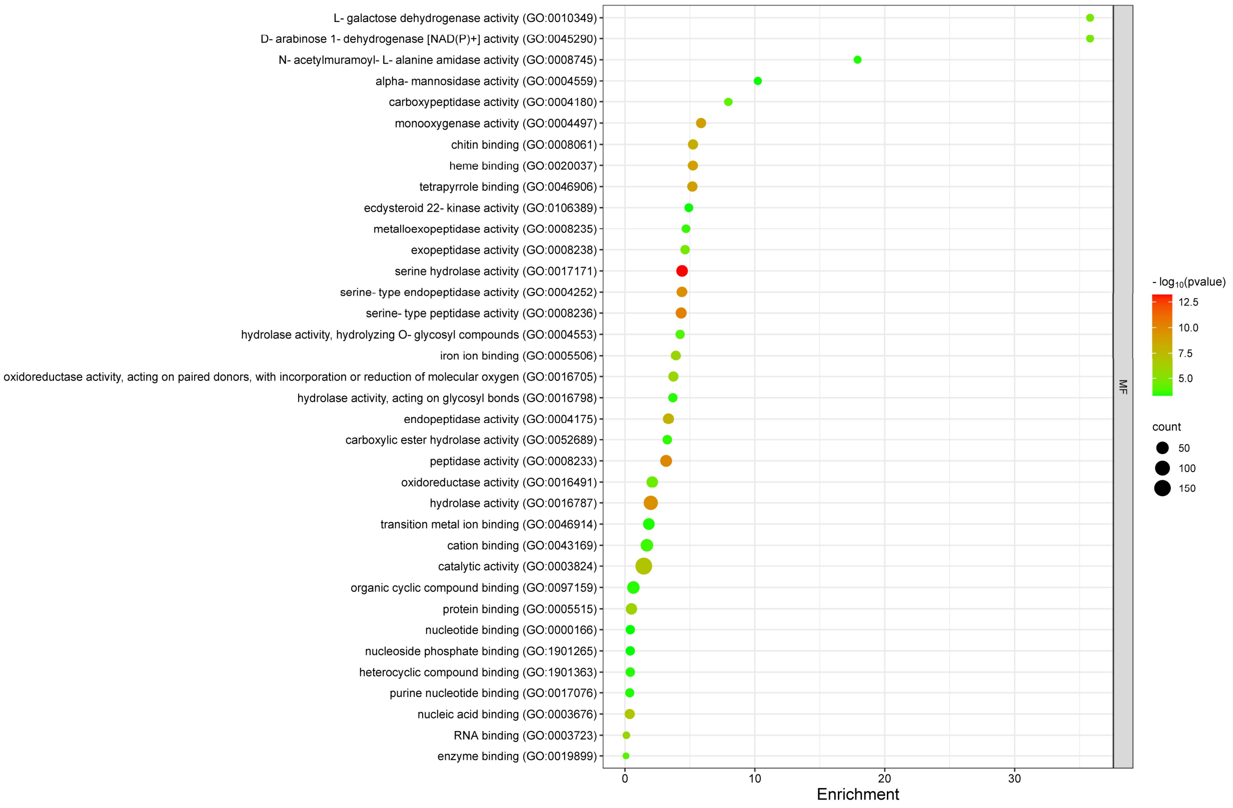
L- galactose dehydrogenase activity (GO:0010349)
D- arabinose 1- dehydrogenase [NAD(P)+] activity (GO:0045290)
N- acetylmuramoyl- L- alanine amidase activity (GO:0008745)
alpha- mannosidase activity (GO:0004559)
carboxypeptidase activity (GO:0004180)
monooxygenase activity (GO:0004497)
chitin binding (GO:0008061)
heme binding (GO:0020037)
tetrapyrrole binding (GO:0046906)
ecdysteroid 22- kinase activity (GO:0106389)
metalloexopeptidase activity (GO:0008235)
exopeptidase activity (GO:0008238)
serine hydrolase activity (GO:0017171)
serine- type endopeptidase activity (GO:0004252)
serine- type peptidase activity (GO:0008236)
hydrolase activity, hydrolyzing O- glycosyl compounds (GO:0004553)
iron ion binding (GO:0005506)
oxidoreductase activity, acting on paired donors, with incorporation or reduction of molecular oxygen (GO:0016705)
hydrolase activity, acting on glycosyl bonds (GO:0016798)
endopeptidase activity (GO:0004175)
carboxylic ester hydrolase activity (GO:0052689)
peptidase activity (GO:0008233)
oxidoreductase activity (GO:0016491)
hydrolase activity (GO:0016787)
transition metal ion binding (GO:0046914)
cation binding (GO:0043169)
catalytic activity (GO:0003824)
organic cyclic compound binding (GO:0097159)
protein binding (GO:0005515)
nucleotide binding (GO:0000166)
nucleoside phosphate binding (GO:1901265)
heterocyclic compound binding (GO:1901363)
purine nucleotide binding (GO:0017076)
nucleic acid binding (GO:0003676)
RNA binding (GO:0003723)
enzyme binding (GO:0019899)
0
10
20
30
Enrichment
MF
- log10(pvalue)
12.5
10.0
7.5
5.0
count
50
100
150

(B)

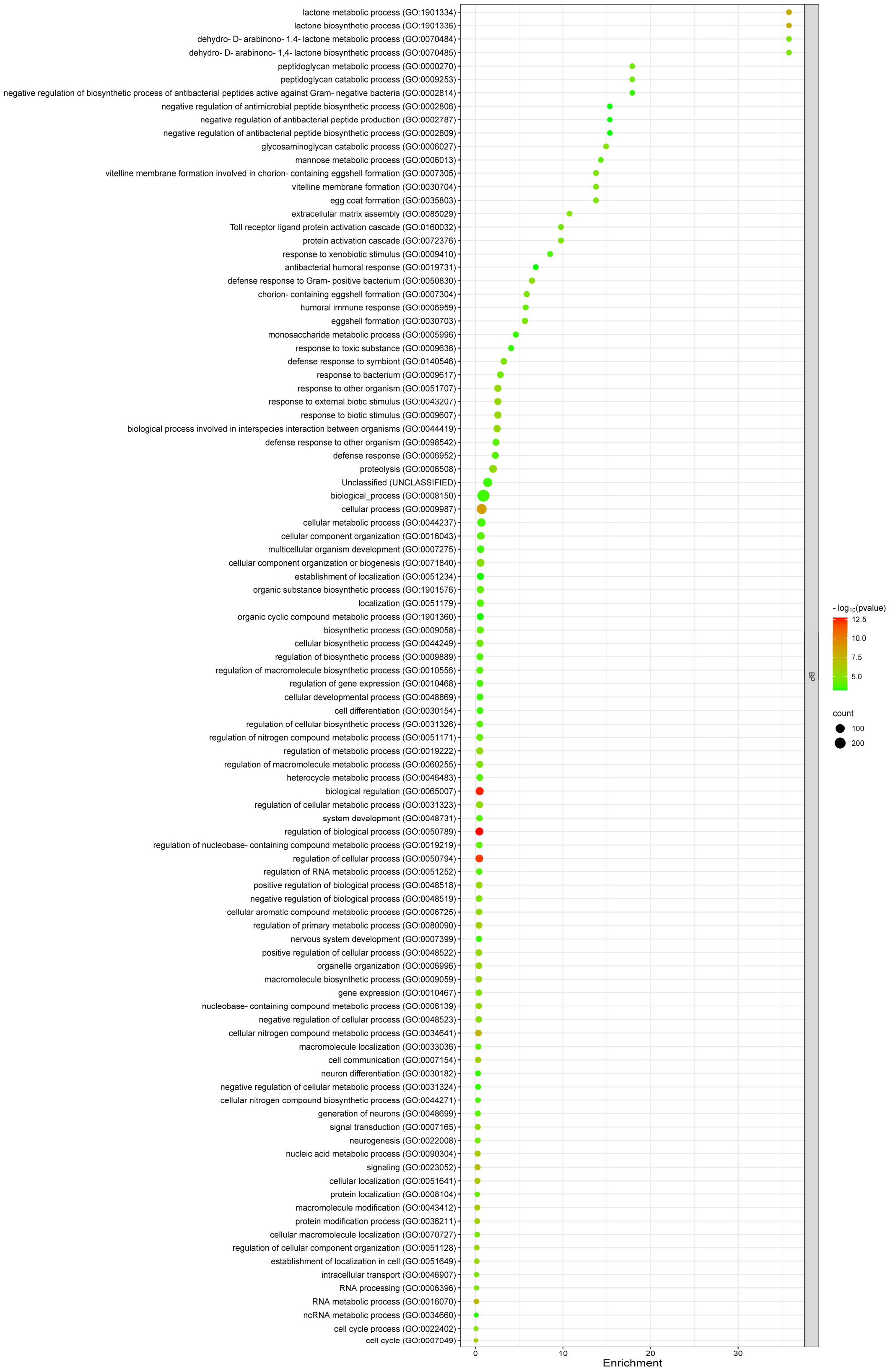

(C)

Figure S1. Functional classification of GO in RNA-seq. (**A**) Functional classification of GO about cellular component (CC); (**B**) Functional classification of GO about molecular function (MF); (**C**) Functional classification of GO about biological process (BP).

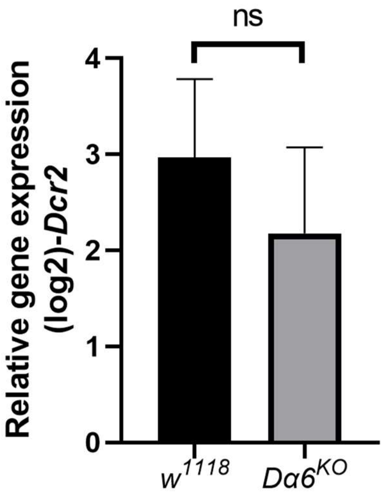

Figure S2. Difference in the expression of Dcr2 in the gene D*α*6 knockout strain flies compared with wild-type flies (w1118) at 6 days after infection with sigma virus.
